# Comment on: “Is There Evidence for the Development of Sex-Specific Guidelines for Ultramarathon Coaches and Athletes? A Systematic Review”

**DOI:** 10.1186/s40798-023-00615-2

**Published:** 2023-09-07

**Authors:** Leonardo Santos Lopes da Silva, Cicero Jonas R. Benjamim

**Affiliations:** 1 School of Physical Education and Sport of Ribeirão Preto, University of São Paulo, Bandeirantes Avenue nº 3900, University Campus - Monte Alegre, Ribeirão Preto, SP 14030-680 Brazil; 2 Department of Internal Medicine, Ribeirão Preto Medical School, University of São Paulo Paulo, Bandeirantes Avenue Avenue nº 3900, University Campus - Monte Alegre, Ribeirão Preto, SP 14049-900 Brazil

**Dear Editor,**

We read with great interest the work done by Kelly [1], who conducted a systematic review (SR) that aimed to review the current knowledge of sex differences in ultramarathon runners and determine if sufficient evidence exists for providing separate guidelines for males and females. First, we want to compliment the author on the relevance of this investigation. In this letter, concerns are raised regarding the methodological aspects of the review, which may have led to misleading conclusions.

The author declared that the review was conducted following the Preferred Reporting Items for Systematic Reviews and Meta-Analysis (PRISMA)-Protocol guidelines [2]. At this point, some mistakes in writing the review results may have been committed since the reference used concerns the writing of SR protocols [2] and not the SR itself [3].

In addition, critical principles of the guidelines for conducting SR were violated:

Despite the author mentioning in the methodology that “The methods were specified in advance and documented in a detailed protocol,” we identified that the SR protocol was not previously registered in a database (e.g., International Prospective Register of Systematic Reviews [PROSPERO]), which reduces the transparency and reproducibility of the study.

Only one reviewer conducted the review, which is at odds with the recommendations of having at least two independent-blinded reviewers between identification and eligibility steps [4]. In this sense, the internal validity of the review is reduced due to possible selection bias of studies [5].

The inclusion and exclusion criteria are outlined according to the specifications that were used to find the selected studies. However, the study flowchart (Figure 1 in the article [1]) only reports the excluded studies as “Ineligible outcome” and “Ineligible population.” It is unclear to the reader why the author excluded such studies, compromising the transparency of how they were included/excluded from the study.

The research question based on the P-population/I-intervention/C-comparison/O-outcome (PICO) model is inaccurate. In addition, the types of studies included in the review were erroneously described in the acronym I-intervention. At this point, the author could create the acronym S-Study Design to provide specific information about the observational studies that will be included in the SR (e.g., cross-sectional/cohort).

The use of the Grading of Recommendations, Assessment, Development, and Evaluations (GRADE) framework is worrying, as some criteria for classifying the certainty of the evidence need to classify the magnitude of the effect of the intervention and the assessment of the risk of bias in the primary studies [6]. Given that most of the included studies are observational and the review does not present a meta-analysis calculation, it is not possible to understand how the reviewer classified the evidence certainty of the primary studies. At this point, no tool for ranking the risk of bias of the primary studies was used, which makes it unclear what quality of evidence the SR conclusions were based on.

In conclusion, it was suggested that sex-specific recommendations for female ultramarathon runners might improve health and performance outcomes. However, the author reports that the evidence base found in the review [1] was of poor quality, making it challenging to infer conclusions regarding the potential benefits of sex-specific approaches to ultramarathon training, racing, nutrition, and recovery.

Due to the lack of methodology information about the questions mentioned above, we invite the author to provide transparent information about that.

## Data Availability

Not applicable.

## Abbreviations

SR: Systematic review
PRISMA: Preferred Reporting Items for Systematic Reviews and Meta-Analysis
PROSPERO: International Prospective Register of Systematic Reviews
PICO: P-population/I-intervention/C-comparison/O-outcome
GRADE: Grading of Recommendations, Assessment, Development, and Evaluations
